# Nanoparticle vaccine based on the pre-fusion F glycoprotein of respiratory syncytial virus elicits robust protective immune responses

**DOI:** 10.1128/jvi.00903-25

**Published:** 2025-08-26

**Authors:** Zhulong Hu, Siyu Tian, Yu Zhou, Yanqun Wang, Yu Li, Senyan Zhang, Peilan Wei, Zhen Zhuang, Luo Ren, Jiao Liu, Na Zang, Rui Yu, Yanbin Ding, Yan Guo, Cai Jing, Hang Chen, Caixia Zhang, Yuanfeng Yao, Chunping Deng, Rui Wei, Peng Zhou, Yongjuan Zou, Dawei Zhao, Shuyun Liu, Meijuan Fu, Xuejun Mo, Guodong Peng, Enmei Liu, Jincun Zhao, Yuanyuan Li, Jing Jin

**Affiliations:** 1 Patronus Biotech Co. Ltd., Guangzhou, China; 2 State Key Laboratory of Biocatalysis and Enzyme Engineering, School of Life Sciences, Hubei University 12563, Wuhan, China; 3 State Key Laboratory of Respiratory Disease, National Clinical Research Center for Respiratory Disease, Guangzhou Institute of Respiratory Health, the First Affiliated Hospital of Guangzhou Medical University 664066, Guangzhou, China; 4 Guangzhou National Laboratory, Bio-Island 612039, Guangzhou, China; 5 Department of Respiratory Medicine, Children's Hospital of Chongqing Medical University, National Clinical Research Center for Child Health and Disorders, Ministry of Education Key Laboratory of Child Development and Disorders, Chongqing Key Laboratory of Pediatrics 159456, Chongqing, China; 6 Pediatric Research Institute, Children's Hospital of Chongqing Medical University, Chongqing, China; 7 Shanghai Institute for Advanced Immunochemical Studies, School of Life Science and Technology, ShanghaiTech University 387433, Shanghai, China; Loyola University Chicago - Health Sciences Campus, Maywood, Illinois, USA

**Keywords:** RSV vaccine, fusion glycoprotein, nanoparticle, Tag/Catcher system, neutralizing antibodies

## Abstract

Respiratory syncytial virus (RSV) is a global public health concern. Currently, RSV vaccines are approved only for use in older adults, while preventing the disease in infants and children, as well as ensuring vaccine durability, remains a significant challenge. The pre-fusion conformation of the RSV fusion (F) glycoprotein is a primary target for vaccine development, as it elicits significantly higher neutralizing antibody titers than the post-fusion form. Here, we conjugated either the first-generation RSV pre-fusion F antigen, DS-Cav1, or the second-generation antigen, Sc9-10, to a computationally designed nanoparticle platform, NPM, via a Catcher/Tag system. Conjugating RSV pre-fusion F to NPM significantly enhanced immunogenicity, stability, and bioactivity compared to display on the I53-50 nanoparticle platform. In a cotton rat challenge model, Sc9-10-NPM vaccine candidates provided effective protection across a wide dosage range, regardless of the adjuvant used. These results support the continued development of this promising nanoparticle-based RSV vaccine candidate.

**IMPORTANCE:**

Respiratory syncytial virus (RSV) is a major cause of severe respiratory illness in infants and young children worldwide, yet few vaccines are approved for use in these vulnerable groups. In this study, we developed a new vaccine candidate based on a second-generation RSV pre-fusion F protein, engineered for improved stability and immune response. This protein was displayed on a specially designed nanoparticle platform to enhance its effectiveness and durability. The vaccine elicited strong immune responses and provided complete protection in preclinical models, even without the use of potent adjuvants that may cause side effects. Importantly, it did not trigger adverse vaccine-enhanced disease (VED). These findings suggest that this vaccine design could offer a safer and more effective way to protect infants and other at-risk populations from RSV. Additionally, the nanoparticle platform may be applicable to vaccines against other infectious diseases.

## INTRODUCTION

Respiratory syncytial virus (RSV) is an enveloped virus that belongs to the genus *Orthopneumovirus* in the family *Pneumoviridae* and order *Mononegavirales*. First isolated from chimpanzees in 1956, the virus can be classified into two major antigenic groups: designated A and B. The surface G glycoprotein exhibits the most significant divergence, with only 53% amino acid identity between subtype A and B viruses (1). RSV infection is generally transmitted through close contact and spreads via aerosolized droplets. RSV is the primary cause of lower respiratory tract disease associated with bronchiolitis and pneumonia in children (2). Nearly all children are infected with RSV by the age of two, and about half experience at least two infections during that period. Globally, RSV infection leads to ~60,000 in-hospital deaths annually in children younger than five years of age. The development of effective interventions is urgently needed to prevent and treat RSV infection (3).

RSV is a non-segmented, negative-sense RNA virus with a 15.2 kb genome. The genome contains 10 sequentially arranged genes encoding 11 viral proteins: the nucleocapsid protein (N), the phosphoprotein (P), the large polymerase (L), the matrix protein (M), an anti-termination factor (M2-1), an RNA regulatory factor (M2-2), two non-structural proteins (NS1 and NS2), and three transmembrane proteins, the glycoprotein (G), the fusion protein (F), and the small hydrophobic protein (SH). The G glycoprotein is a highly glycosylated protein that mediates RSV virions’ attachment to the host cellular membrane. After attachment, the fusion (F) glycoprotein triggers the fusion of the viral envelope and the cell membrane, followed by the release of helical ribonucleoprotein complex (RNP) into the host cell cytoplasm (4, 5). The F glycoprotein is an attractive vaccine candidate since it is the principal target of RSV-neutralizing antibodies in human sera (6). The F glycoprotein is a trimeric class I fusion glycoprotein that undergoes proteolytic cleavage to produce two disulfide-linked polypeptides, F1 and F2. Like other viral fusion glycoproteins, RSV F experiences an irreversible structural rearrangement during the fusion process, and it refolds from the labile pre-fusion conformation to the stable post-fusion conformation. While the post-fusion state of RSV F has been shown to induce only modest increases in neutralizing antibodies, the pre-fusion state (preF) can induce robust production of neutralizing antibodies (7). Scientists in the field have spent decades obtaining stable pre-fusion F mutations. Peter D. Kwong’s group first introduced a trimer-stabilization mutation by a disulfide between residues 155 and 290 (“DS”) and cavity-filling mutations S190F and V207L (“Cav1”), collectively termed “DS-Cav1,” to sustain the pre-fusion conformation of RSV F glycoprotein. The DS-Cav1 surprisingly elicited high-titer protective responses against RSV in mice and macaques (8). Based on DS-Cav1, the second-generation RSV pre-fusion F immunogens were further optimized. In these immunogens, the F subunits (F1 and F2) were genetically linked, the fusion peptides were deleted, and the interprotomer movements were stabilized by the DS-Cav1 recombinants. Among these, a single-chain variant termed Sc9-10 DS-Cav1 A149C Y458C (referred to as SC9-10 in our article for conciseness) exhibited enhanced immunogenicity, stability, and production profile relative to the first-generation DS-Cav1 (9).

The well-defined structural and biological characteristics of the RSV pre-fusion F glycoprotein make it an ideal target for vaccine development. Application of the RSV pre-fusion F subunit as a vaccine candidate has elicited robust protective immune responses in multiple clinical trials. Subsequently, both GSK and Pfizer announced RSV vaccine candidates based on the pre-fusion F subunit design. Clinical trials demonstrated 94.1% and 85.7% efficacy in older adults, respectively (10, 11). Both vaccines were approved by the FDA for marketing in 2023. In recent years, recombinant self-assembling nanoparticle (NP) carriers have undergone rapid development and are considered a powerful platform for antigen display due to their ability to induce more robust and durable humoral and cellular immunity than monomeric antigens. Several platforms, such as ferritin, AP205, Qβ, mi3, and I53-50, have been used to present viral surface glycoproteins derived from influenza virus, Epstein-Barr virus, and SARS-CoV-2 (12, 13). Repetitive presentation of antigens on NP carrier significantly increases the immunogenicity of the antigen and elicits potent neutralizing antibody production. Displaying RSV pre-fusion F, stabilized as DS-Cav1, on I53-50 NPs through fusion expression with the I53-50A subunit resulted in a tenfold more potent neutralizing antibody response compared to trimeric DS-Cav1 (14). In this study, we conjugated either the first-generation RSV pre-fusion F antigen, DS-Cav1, or the second-generation antigen, Sc9-10, to a computationally designed nanoparticle platform mi3 (abbreviated as NPM in this article), which is based on the 2-dehydro-3-deoxy-phosphogluconate (KDPG) aldolase. Conjugation was achieved via a Catcher/Tag system as previously reported (15). Conjugating RSV pre-fusion F to NPM significantly enhanced its immunogenicity, stability, and bioactivity compared to preF displayed on the I53-50 carrier. This construct holds promise as a competitive vaccine candidate, and the Catcher/Tag-based NPM design could be adopted as a universal platform for future vaccine development.

## MATERIALS AND METHODS

### Expression and purification of recombinant pre-F glycoprotein antigens and nanoparticle carriers

**Fig 1:**
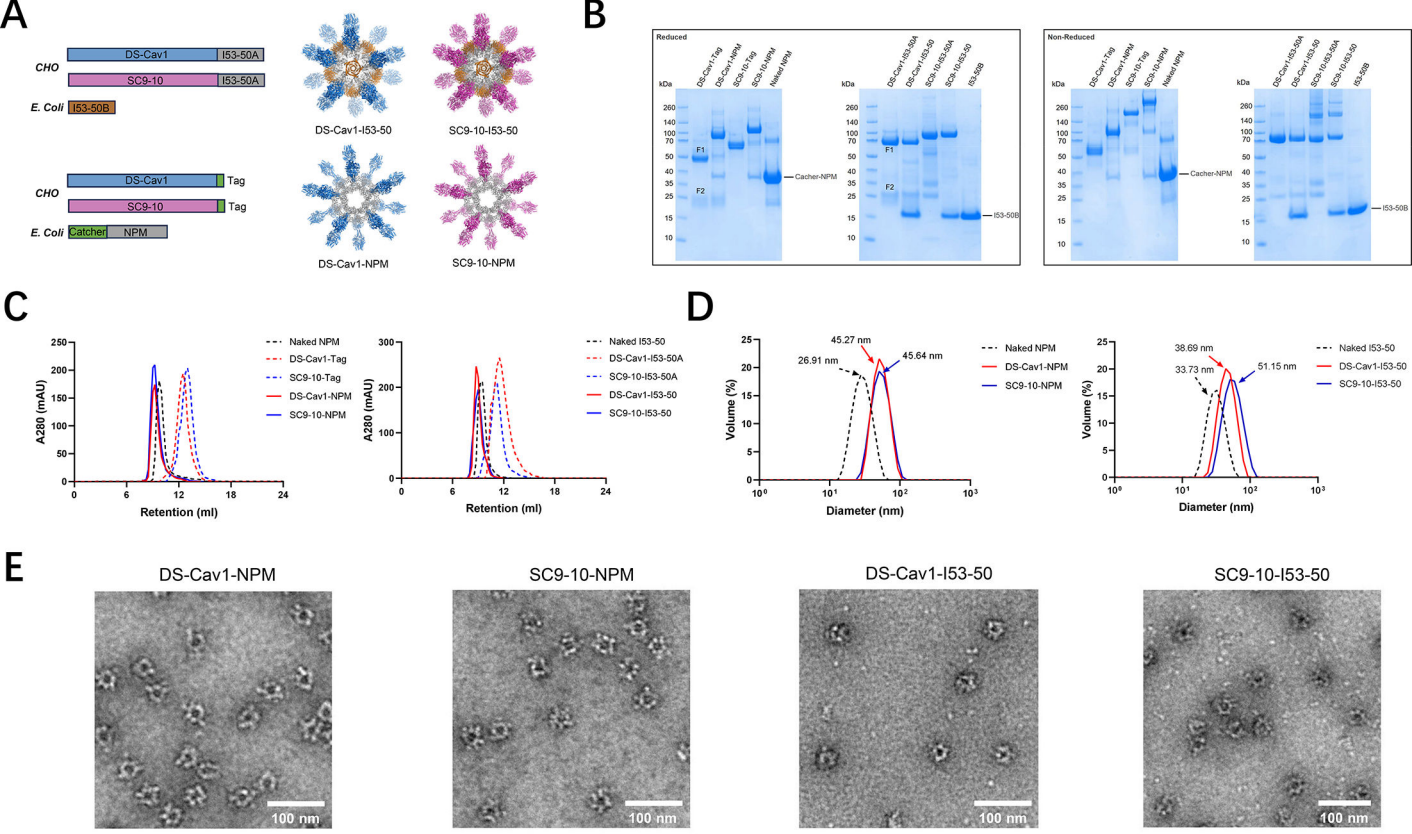
Development and characterization of different RSV preF-NPs. (**A**) Schematic representations of the preF antigens and NP carriers. SP = signal peptide. (**B**) SDS-PAGE results of the preF antigens and preF-NPs under reduced or non-reduced conditions. (**C**) Size exclusion chromatography (SEC) results of the preF antigens, preF-NPs, and naked NPs on a Superdex-200 increase 10/300 Gl column. (**D**) Dynamic light scattering (DLS) of the preF-NPs and naked NPs. The arrows indicate the average measurement of diameter (*n* = 3 individual measurements). (**E**) Negative-staining EM of the pre-F-NPs.

RSV preF (aa 26-513) was engineered with a signal peptide for secretion from mammalian cells and a C-terminal Tag from the isopeptide linkage system or the I53-50A component to enable antigen display on the corresponding NP carriers (Fig. 1A). The preF-Tag and preF-I53-50A constructs were codon-optimized and expressed in a stable Chinese hamster ovary (CHO) cell line as a secreted protein. From the clarified supernatant, high-purity fusion proteins were produced using a combination of orthogonal chromatographic methods on AKTA systems (Cytiva) and buffer exchanged into the final formulation buffer (20 mM Tris-HCl, 150 mM NaCl, pH 7.4) using a tangential flow filtration (TFF) system (Cobetter) equipped with Pellicon 2 Biomax 10 kDa MWCO membrane (Merck-Millipore). The Catcher from the isopeptide linkage system was genetically fused to a flexible linker, followed by the subunit of the selected NP carrier, including a computationally designed NP, abbreviated as NPM (nanoparticle mi3) in this study, based on 2-dehydro-3-deoxy-phosphogluconate (KDPG) aldolase, as previously described (15). This Catcher enables the formation of an isopeptide bond with the Tag. For preF-I53-50A, it self-assembles with the I53-50B component into an NP (16). These Catcher-NPs (naked NPs) were codon-optimized and expressed in *Escherichia coli* BL21 (DE3). Biomass was bulked up in an XDR-50 MO single-use fermenter (Cytiva) and harvested in Sorvall Lynx 6000 centrifuges (Thermo Fisher Scientific). Intracellular proteins were first released using an AH-PILOT high-pressure homogeniser (ATS) and then clarified by a series of centrifugations. Catcher-NPM and I53-50B were produced using a combination of orthogonal chromatographic methods on AKTA systems (Cytiva) and buffer exchanged into final formulation buffer (20 mM Tris-HCl, 25% sucrose w/v, pH 9.0, used for Catcher-NPM and 50 mM Tris-HCl, 300 mM NaCl, 0.75% CHAPS w/v, pH 7.4, used for I53-50B) using a TFF system (Cobetter) equipped with Pellicon 2 Biomax 300 kDa MWCO membrane (Merck-Millipore). For conjugation, the Catcher-NPM and preF-Tag were mixed at a 1:6 molar ratio and incubated for 24 hours at 4°C. I53-50B and preF-I53-50A were mixed at a 3:1 mass ratio and incubated for 2 hours at room temperature (RT). Uncoupled preF-Tag and excessive I50-53B were removed from the conjugated NP by size-exclusion chromatography (SEC) using a HiLoad 16/600 Superdex 200 pg column (Cytiva), pre-equilibrated with 20 mM Tris-HCl, 25% sucrose w/v, pH 7.4, on an AKTA system (Cytiva). After separation, the conjugated NPs were analyzed on sodium dodecyl sulfate polyacrylamide gel electrophoresis (SDS-PAGE).

### Production of MF59-biosimilar adjuvant

Similar to MF59 manufacturing, the process involves dispersing sorbitan trioleate in the squalene phase and polysorbate 80 in the aqueous phase, followed by high-speed mixing using the SMART LAB homogenizer (FLUKO) to form a coarse emulsion. The coarse emulsion then passed through a high-pressure ATS-AH pilot homogenizer (ATS Engineering Limited) to produce a fine emulsion. The emulsion is filtered through a sterilized filter, yielding MF59-bio at a droplet size of around 145–165 nm.

### SDS-PAGE

For SDS-PAGE, NuPAGE 4–12% Bis-Tris Midi Gel (Thermo Fisher Scientific) was used for all the analyses in this study. Before loading, protein samples were mixed with NuPAGE LDS Sample Buffer (Thermo Fisher Scientific) with or without 5 mM DTT and denatured at 95°C for 5 minutes (min). Electrophoresis was performed at 150 V for 1 hour in an XCell4 SureLock Midi-Cell (Thermo Fisher Scientific) filled with 1× NuPAGE MES SDS Running Buffer (Thermo Fisher Scientific). Gels were then stained with InstaBlue Protein Stain Solution (APExBIO) and imaged using the Amersham ImageQuant 800 system (Cytiva).

### BCA assay

The bicinchoninic acid (BCA) assay was performed using Pierce BCA Protein Assay Kit (Thermo Fisher Scientific) for total protein quantification according to the manufacturer’s instructions. Briefly, a series of bovine albumin standards were diluted to 0.025–1.5 mg/mL using the same diluent as the sample. Twenty-five microliters of each standard and sample were pipetted into a Nunc Microwell 96F plate (Thermo Fisher Scientific) in triplicate and incubated with working reagent at 37°C for 30 min. Absorbance at 562 nm was measured using an Infinite 200 PRO plate reader (Tecan) and plotted to construct a standard curve for sample evaluation.

### Dynamic light scattering (DLS)

Dynamic light scattering (DLS) was performed using Malvern Zetasizer Lab (Malvern) equipped with a 633 nm He-Ne laser. Size measurements were operated at an angle of 90°, and data were collected and analyzed using ZS XPLORER (Malvern). Approximately 1 mL of the sample (at 0.25 mg/mL) was measured in a DTS0012 disposable polystyrene cuvette (Malvern) at a controlled temperature of 25°C. Viscosity and refractive index at 25°C have been adjusted accordingly. Testing of each sample was repeated three times.

### Negative staining electron microscopy (EM)

Three to four microliters of preF-NP sample at a concentration of 100 ng/µL was adsorbed onto a glow-discharged carbon-coated copper grid for 1 min. The grid was then washed with Milli-Q water and blotted dry. Negative staining was performed with 0.75% uranyl formate for 1 min. Electron micrographs of preF-NP were recorded using an FEI Tecnai 12 transmission electron microscope (Thermo Fisher Scientific) equipped with an Orius SC200 CCD camera operating at 120 kV.

### Immunization

For naïve mice immunization, six- to eight-week female BALB/c mice purchased from Charles River, housed in specific pathogen-free (SPF) environments, were vaccinated via the intramuscular (i.m.) route, depending on the design of the experiment, using a prime-boost regime (prime on day 0 and boost on day 14). preF-NP immunogens were formulated with PBS, Alhydrogel, or MF59-bio adjuvant and mixed thoroughly before injection. Blood samples were collected on day 28, and sera were obtained from whole blood by leaving samples overnight at 4°C to clot, followed by 10 min centrifugation at 16,000 × *g* at RT. Sera were pipetted into fresh Eppendorf tubes and frozen until further analysis.

### Cell line and viruses

HEp-2 (ATCC CCL-23) cells were cultured in Dulbecco’s modified Eagle’s medium (DMEM) containing 10% fetal bovine serum (FBS) with 100 U/mL penicillin–streptomycin (Gibco). RSV strain A2 (ATCC) and RSV strain B18537 (ATCC) were produced in HEp-2 cells.

### IgG endpoint ELISA

Anti-preF total IgG endpoint titer of serum collected from immunized animals was determined by indirect ELISA assay. Ninety-six-well Nunc MaxiSorp plates (Thermo Fisher Scientific) were coated with pre-F at 100 ng per 50 µL/well or NPM at 25 ng per 50 µL/well overnight at 4°C. After washing two times with 0.05% Tween 20 in PBS (PBST), the plates were blocked with Blocker Casein in PBS (Thermo Fisher Scientific) at 200 µL/well for 1 hour at RT before washing two times again with PBST. Serially diluted sera were applied to each well for 1 hour at RT. The plate was then washed four times with PBST, followed by incubation with a 1:5000 dilution of goat anti-mouse IgG conjugated with HRP (Abcam) for 1 hour at RT. After six additional washes with PBST, the plates were developed using tetramethylbenzidine (TMB) (Thermo Fisher Scientific, USA) for 10 min at RT. Subsequently, 1 N HCl was added to stop the reaction, and the absorbance was recorded at 450 nm–620 nm. The endpoint titer is defined as the X-axis intercept of the dilution curve at an absorbance value (± two standard deviations or 0.15, whichever is higher) greater than the optical density (OD) for a naïve serum.

### Competitive ELISA (mice)

The levels of serum antibodies specific for preF were determined using a competitive ELISA. Briefly, 96-well ELISA plates were coated with D25 at 200 ng per 50 µL/well overnight at 4°C. Immunized mouse serum was serially diluted threefold and mixed (1:1 ratio) with 0.1 µg/mL pre-F, and then, 100 µL of the mixture was transferred to a D25-coated plate and incubated at RT for 1 hour. HRP Anti-6X His tag antibody (Abcam) was added for another 1 hour at RT, followed by TMB substrate. The serum neutralizing antibody titers were determined as described above.

### Live virus neutralization assay (mice)

The levels of neutralizing antibodies against RSV strain A2 and strain B18537 were measured with Hep-2 cells. In short, 10,000 cells/well were plated on 96-well plates overnight at 37°C in a CO_2_ incubator. On the day of the experiment, 12.5 µL of immunized mouse serum was added to 87.5 µL of DMEM, serially diluted fourfold, mixed with 100 TCID50/75 µL of RSV strain A2 or strain B18537, and incubated at 37°C for 2 hours. During incubation, 96-well microplates were washed three times with PBS, and then, the mixtures were transferred to the cell plate for an additional 2 hours of incubation. After 5 to 7 days, observation of cytopathic effect (CPE) was performed with an inverted microscope, and the serum virus neutralization titer (VNT50) was defined as the reciprocal value of the sample dilution that showed a 50% protection of virus growth.

### Cotton rat immunization and RSV challenge

Two challenge studies were performed in this study. In brief, male SPF-grade cotton rats were immunized on day 0 and day 21 intramuscularly with candidate vaccines and controls. In challenge experiment #1, animals were vaccinated with 60 µg of SC-9-10-NPM with and without adjuvant and 60 µg of control vaccine Arexvy. The control groups (PBS control and healthy animal control) were included. In challenge experiment #2, animals were immunized with 3 µg, 15 µg, 30 µg, or 60 µg of SC-9-10-NPM formulated either without adjuvant or with MF59-bio. The PBS control, healthy animal control, and the group receiving inactivated RSV formulated in aluminum hydroxide were included. For both experiments, serum was collected on day 41 to measure neutralizing antibodies against RSV strain A2 and RSV strain B18537. To assess efficacy, cotton rats were challenged intranasally on day 42 with 1 × 10^6^ plaque-forming units (PFU) of RSV strain A2. Animals were sacrificed on day 46, and the lung samples were divided into two parts. The right lung was quickly frozen in HBSS solution with a volume of 10 times the tissue weight, while the left lung was perfused with 0.8 mL of 4% paraformaldehyde and fixed in 4% paraformaldehyde. The turbinate bone tissue (for both experiments) and trachea tissue (for experiment #1 only) were quickly frozen in HBSS solution with a volume of 10 times the tissue weight and stored at −80°C until assayed.

### Neutralization antibody titers in serum measured by microneutralization (cotton rat)

Serum samples were heat-inactivated at 56°C for 30 min. Initially, the serum was diluted 1:20 in DMEM, followed by a series of threefold dilutions, resulting in a total of eight dilution points. Simultaneously, a new 96-well microplate was prepared, where the virus was twofold serially diluted in 100 μL/well for virus back titration verification. Fifty microliters of serially diluted serum and 50 µL of diluted virus (200 TCID50) were added to each well of a 96-well microplate and incubated for 2 hours at 37°C in a 5% CO_2_ incubator. After incubation, Hep-2 cells were inoculated into the test plate and the back titration plate at a density of 25,000 cells per well. The cell control group (cells without virus infection) and the virus control group (cells infected with virus, receiving no other treatment) were established in parallel. Cells were cultured in an incubator at 37°C with 5% CO_2_ for five days. Eighty percent acetone was added to fix the cells at 4°C for 15 min, and the plate was then air-dried. Primary and secondary antibodies were sequentially added to each well and incubated at 37°C for 1 hour. Finally, TMB premixed solution was added, followed by 1% HCl to stop the reaction. A microplate reader was used to read the absorbance at 450 nm.

The neutralization titer (NT50) of the serum was calculated and data analyzed using GraphPad Prism software based on the equation below.


$$\%\text{Antibody activity}=100-\left(\frac{{\text{OD}}_{\text{sample}-\text{cell control}}}{{\text{OD}}_{\text{virus control}-\text{cell control}}}\right)\times 100$$

The curve fitting method utilized a variable slope with a log (inhibitor) versus response model.

### RSV titers in tissues (cotton rat)

Cotton rat lungs, turbinate bones, and trachea homogenates were generated and used for viral detection. RSV titers in tissue were detected by plaque assay. HEp-2 cells were added to the 12-well cell plates and cultured in a CO_2_ incubator at 37°C overnight to obtain monolayer cells. The ground tissue homogenate was mixed and subsequently subjected to centrifugation. The supernatant was collected and diluted eight times with the experimental medium using three gradients (50 µL supernatant + 350 µL DMEM). The resulting mixture was incubated in a cell incubator at 37°C for 4 hours to facilitate complete viral adsorption. Following incubation, paraformaldehyde was used for fixation. Primary and secondary antibodies were then added to each well and incubated for the appropriate time. A color solution comprising components A and B was prepared and added to each well, followed by incubation in the dark for 15 min. After color development, ddH2O was added to halt the reaction, and the wells were then washed and dried at 37°C. The resulting images were scanned, and plaque counts were recorded to calculate the viral titer in the sample, expressed as the logarithm of the plaque count per gram of tissue homogenate.

### Lesions of lung tissue measured by HE staining (cotton rat)

After perfusion, the lung tissue was fixed in 4% paraformaldehyde for a minimum of 24 hours. Following fixation, the tissue underwent dehydration. Subsequently, the sample was embedded in paraffin, sliced into sections with a thickness of 4 µm, and placed in a staining rack. The rack was then incubated in an oven at 60–65°C for 1 hour to allow the paraffin to melt into a transparent liquid state. After melting, the staining rack was cooled at room temperature for 10 min, followed by HE staining and sealing of the lung sections, before finally being scored by an evaluator who was blinded to the group assignment.

### Quantification and statistical analysis

GraphPad Prism 7.00 was used for statistical analysis of the data. The Kruskal–Wallis test was used to analyze the statistical difference of neutralizing antibody titers, lung tissue, and turbinate bone viral titers. Ordinary one-way ANOVA was used to analyze the statistical difference of lung tissue pathology. *P*-values less than 0.05 were considered significant (*0.01 < *P* < .05, **0.001 < *P* < .01, ***0.0001 < *P* < .001, and ****P < 0.0001).

## RESULTS

### Design and preparation of RSV preF-conjugated nanoparticles

Nanoparticle-based immunogens offer potential benefits for boosting vaccine-induced immune responses, as studies have indicated that highly organized and repetitive protein epitopes can effectively engage B cells through cross-linking of their receptors (17). In this study, we focus on the extracellular domain of RSV preF glycoprotein as the immunogen. Two stable RSV preF mutants, DS-Cav1 and Sc9-10, containing a C-terminal T4 fibritin trimerization motif (foldon), were chosen to display on the nanoparticle mi3 (NPM), based on a split protein Tag/Catcher technology as previously described (15). The DS-Cav1 and Sc9-10 ectodomains were further displayed on the I53-50 platform by genetically fusing them to the trimeric I53-50A subunit. The resultant conjugated NPs were designated as DS-Cav1-NPM, Sc9-10-NPM, DS-Cav1-I53-50, and Sc9-10-I53-50, respectively. NPM is computationally designed based on the 2-dehydro-3-deoxy-phosphogluconate (KDPG) aldolase, which self-assembles into a dodecahedral 60-mer particle. Several mutations were introduced to promote the assembly process and enhance the homogeneity of the particles. I53-50 is an icosahedral nanoparticle designed computationally by the co-assembly of two components: 12 pentameric I53-50B subunits and 20 trimeric I53-50A subunits (16).

The RSV preF recombinant proteins were expressed in CHO stable cell lines, and NP carriers were expressed in *E. coli* BL21 (DE3) cells, respectively. DS-Cav1-Foldon-Tag, Sc9-10-Foldon-Tag, DS-Cav1-I53-50A, and Sc9-10-I53-50A, as well as the conjugated preF-NPs, achieved a high purity, with the endotoxin levels below 10 EU/mL. The DS-Cav1 recombinants exhibited two distinct bands (F1 and F2) due to cleavage during protein maturation, whereas the Sc9-10 recombinants displayed only a single band, attributed to the substitution of the furin protease cleavage site. The RSV preF recombinant proteins were successfully displayed on the NPM or I53-50 since the conjugated RSV preF migrated to a higher molecular mass on SDS-PAGE as expected. All the RSV preF-NPs were uniform and pure, demonstrated by clear bands (conjugated and unconjugated subunit bands in NPM; subunit A and B bands in I53-50) in SDS-PAGE (Fig. 1B), a single symmetrical peak in the size exclusion chromatography (SEC) (Fig. 1C), and a uniform distribution of particle sizes in a dynamic light scattering (DLS) assay (Fig. 1D). Not all NPM subunits can conjugate with the DS-Cav1 or Sc9-10, presumably because of steric hindrance on the particle surface. Through densitometry analysis, the overall conjugation efficiency of the constructs utilizing the Catcher/Tag system was determined to exceed 70%. On the other hand, the assembly efficiency of DS-Cav1-I53-50 and Sc9-10-I53-50 is theoretically at 100% since the formation of the I53-50 nanoparticle requires the complete assembly of both I53-50A and I53-50B components. Negative-stain electron microscopy further confirmed the morphology of the NPs, revealing that the DS-Cav1 and Sc9-10 antigens were densely coated and were uniformly distributed across the surface of the particles (Fig. 1E). These results indicate that both NPM and I53-50 served as suitable platforms for efficient immunogen display, and such highly organized and repetitive morphologies offer the potential to induce robust immune responses.

### Antigenicity, immunogenicity, and biophysical characterization of RSV preF-conjugated nanoparticles

**Fig 2:**
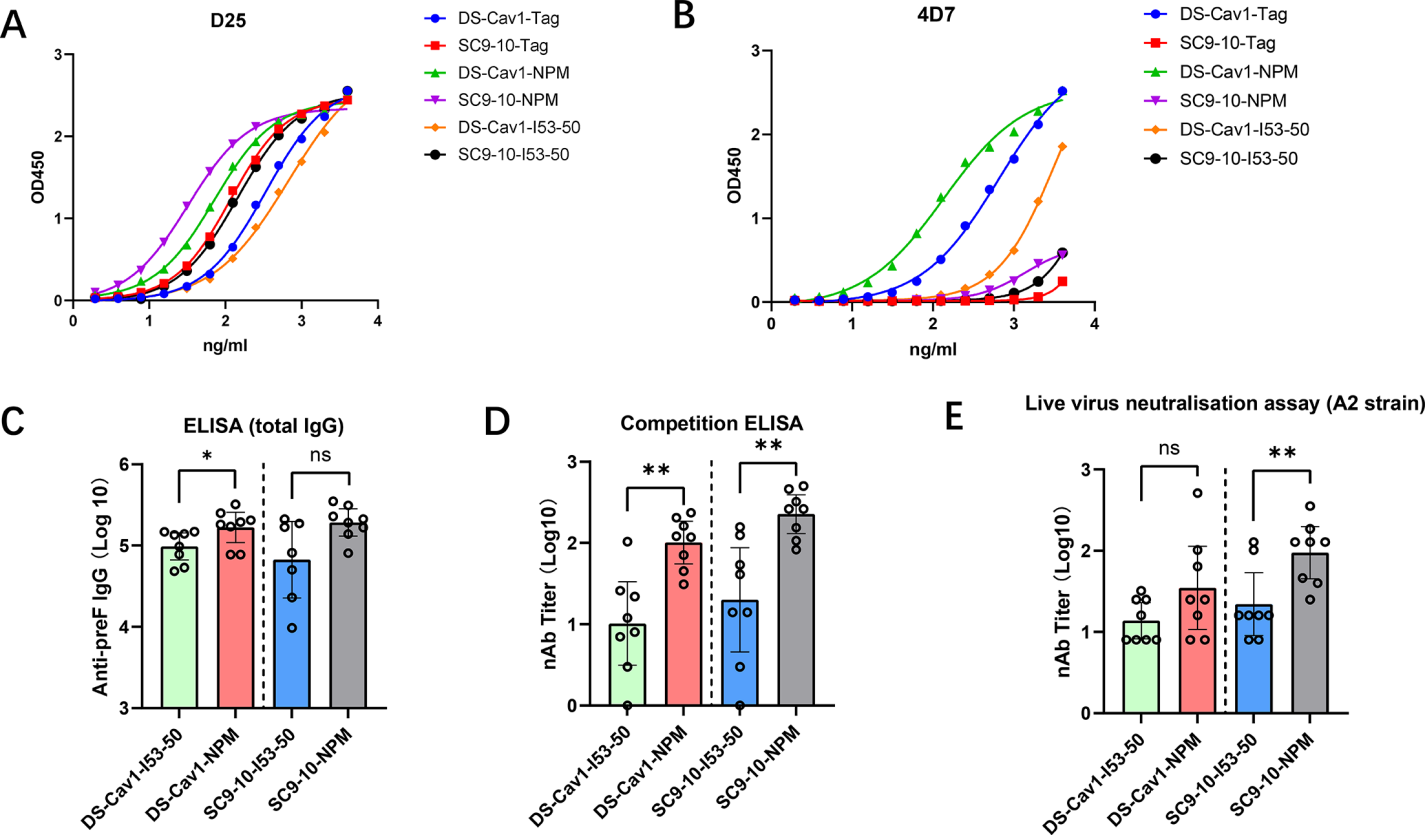
Immunological characteristics and immunogenicity of different RSV preF-NPs. The ELISA profiles of RSV preF and preF-NPs binding with the pre-fusion-specific antibody D25 (**A**) and the post-fusion-specific antibody 4D7 (**B**). BALB/c mice (*n* = 8 per group) were immunized intramuscularly twice with 5 µg of different preF-NPs on day 0 and day 14, respectively. All preF-NPs vaccines were formulated with Alhydrogel, and the serum was collected on day 28. (**C**) Total IgG titers against antigen preF were measured by endpoint ELISA. GMT ± 95% CI is shown. (**D**) Neutralizing antibody titers were measured by competitive ELISA. GMT ± 95% CI is shown. (**E**) Neutralizing antibody titers against RSV A2 strain were measured by live virus neutralization assay. GMT ± 95% CI is shown. Mann-Whitney test was performed, ns = not significant, *P < 0.05, **P < 0.01, ***P < 0.001.

We next expressed and purified two typical monoclonal antibodies, D25 and 4D7, which can specifically recognize the pre-fusion and post-fusion conformation of RSV F glycoprotein, respectively (18, 19). Enzyme-linked immunosorbent assay (ELISA) profiles showed that both DS-Cav1 and Sc9-10 bound robustly to the prefusion-specific antibody D25 (Fig. 2A). It indicates that the mutations introduced in DS-Cav1 and Sc9-10 successfully stabilized their structural conformation at the pre-fusion state. Iterative structural-based improvement on Sc9-10 additionally increases antigenic stability, as it shows a stronger affinity with D25 than DS-Cav1 and a weaker interaction with the post-fusion-specific antibody 4D7 (Fig. 2B). Analogous to the soluble trimeric subunits, DS-Cav1- or Sc9-10-conjugated nanoparticles also effectively bound to the pre-fusion-specific antibody D25, suggesting that the pre-fusion conformation of DS-Cav1 and Sc9-10 was retained intact on the conjugated NPs. Notably, D25 antibody exhibited higher binding affinity for the DS-Cav1- or Sc9-10-conjugated NPM compared to their soluble trimeric subunits or their conjugates to I53-50 NP, indicating that NPM platform may further enhance the antigenic stability of the soluble trimeric preF antigen (Fig. 2A). Overall, multivalent display on nanoparticles could effectively improve the antigenicity of RSV preF trimeric soluble subunits. Among the constructs tested, Sc9-10-conjugated NPM shows the highest affinity with D25 and could be developed as an attractive vaccine candidate to prevent RSV infection.

Next, we conducted a comparative analysis of the immunogenic properties of four RSV preF-conjugated nanoparticles. Mice were stratified into four groups and immunized with 5 µg of each of the following nanoparticles on days 0 and 14: DS-Cav1-I53-50, DS-Cav1-NPM, Sc9-10-I53-50, and Sc9-10-NPM. All nanoparticles were adjuvanted with Alhydrogel, and serum samples were collected on day 28 for subsequent analysis. As expected, endpoint ELISA measurements of total IgG titers against the RSV preF antigen revealed robust humoral immune responses induced by all four nanoparticle formulations. Notably, mice immunized with DS-Cav1-NPM and Sc9-10-NPM nanoparticles exhibited elevated binding antibody responses compared to those vaccinated with DS-Cav1-I53-50 and Sc9-10-I53-50 nanoparticles (Fig. 2C). We conducted a competitive ELISA to assess the neutralizing antibody titers (nAb) in serum samples that competitively bind to neutralizing epitopes with the D25 antibody. Mice immunized with DS-Cav1-NPM and Sc-9-10-NPM nanoparticles elicited significantly higher D25-competitive neutralizing antibody titers than DS-Cav1-I53-50 and Sc-9-10-I53-50 (Fig. 2D). In addition, all constructs with SC9-10 induced higher nAb titers than their DS-Cav1 counterpart, confirming the antigenic superiority of the SC9-10 design. A live virus neutralization assay against the A2 strain of RSV was also performed on the same serum samples. A similar trend to the competition ELISA was observed: animals immunized with the DS-Cav1 and Sc9-10 conjugated with NPM exhibited higher nAb titers, and the SC9-10 constructs generally induced higher nAb response than DS-Cav1 (Fig. 2E).

Lastly, we performed extensive stability monitoring of these nanoparticle vaccine candidates under diverse temperature conditions over a prolonged period. The DS-Cav1- and Sc9-10-conjugated NPM and I53-50 nanoparticles were stored at 4°C, 25°C, and 37°C for up to four weeks, or at −80°C for up to four months. SDS-PAGE analysis demonstrated that all four nanoparticles exhibited a good stability profile, showing no signs of degradation after storage at 4°C for four weeks or at −80°C for six months. However, irreversible degradation began to appear in the DS-Cav1- and Sc9-10-conjugated I53-50 nanoparticles as early as the third day of storage at 25°C or 37°C. The overall stability of preF-conjugated NPM is therefore better than that of I53-50, at least with the selected antigen in our study (Fig. S1). Sc9-10-NPM is then considered a highly promising vaccine candidate for further biophysical analysis. Sedimentation velocity analytical ultracentrifugation (SV-AUC) demonstrated a uniform peak at 5,379 kDa (Fig. S2A). Differential scanning calorimetry (DSC) analysis showed a double-peak thermograph with the first and second melting temperatures determined at 62.3°C and 82.0°C, respectively (Fig. S2B). Biolayer Interferometry (BLI) analysis demonstrated the binding of pre-fusion-specific antibody D25 again, as well as the quaternary-specific antibody AM14, confirming the pre-fusion and the trimeric status of the displayed antigen (Fig. S2C).

### Immunogenicity, dosing, and adjuvants of Sc9-10-NPM in mice

**Fig 3:**
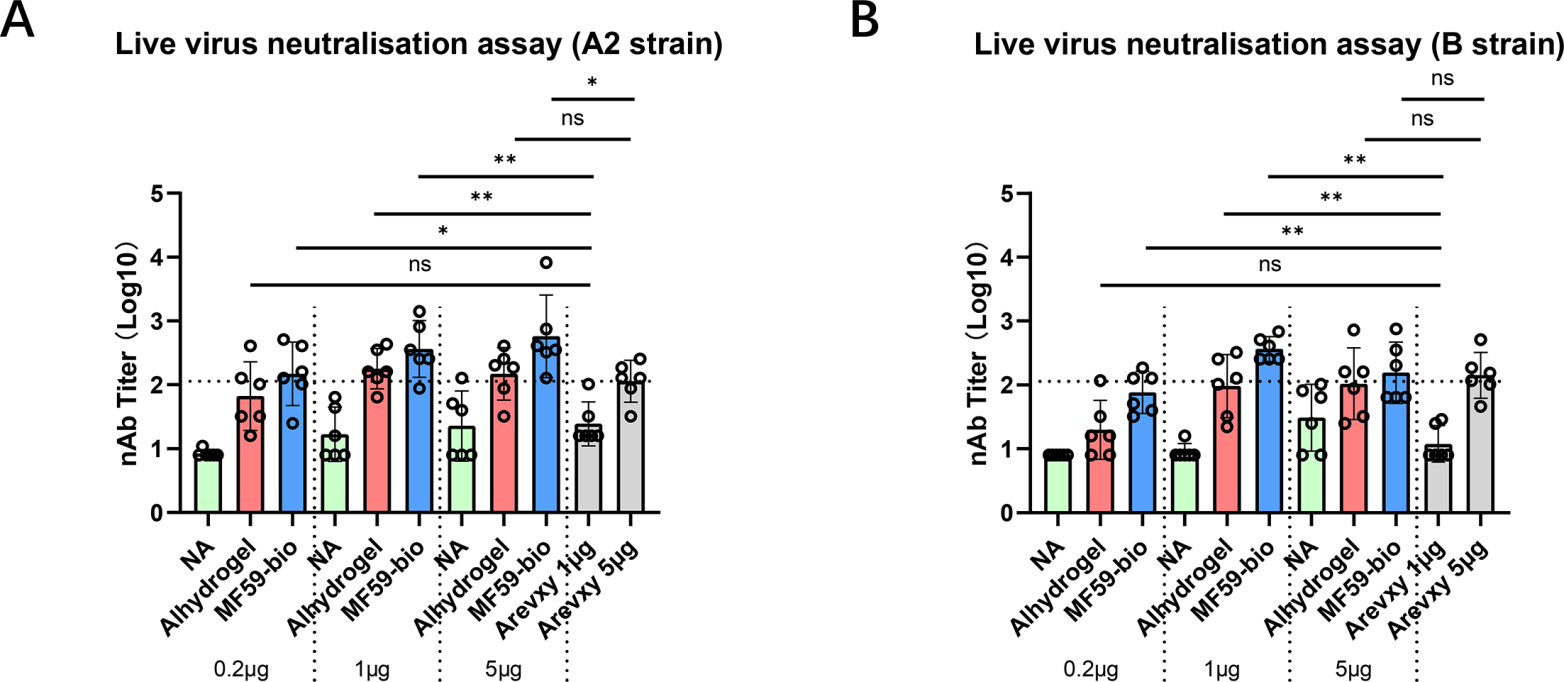
Immunogenicity of SC-9-10-NPM formulated with different adjuvants. BALB/c mice (*n* = 6 per group) were immunized on day 0 and day 14 intramuscularly with 0.2 µg, 1 µg, or 5 µg of SC-9-10-NPM and 1 µg or 5 µg of Arevxy, respectively. SC-9-10-NPM was formulated with PBS (NA group), Alhydrogel, or MF59 biosimilar. Serum was collected on day 28. Neutralizing antibody titers against RSV A2 strain (**A**) and B strain (**B**) were measured by live virus neutralization assay. GMT ± 95% CI is shown. The dotted line represents the antibody titer level by 5 µg of Arevxy. Kruskal–Wallis test with Dunn’s multiple comparison test was performed, ns = not significant, *P < 0.05, **P < 0.01.

Sc9-10-NPM was further evaluated for its immunogenicity in naïve BALB/c mice at varying dosages with and without adjuvants. After two immunizations at day 0 and day 14, day 28 serum was tested for antibody response (Fig. 3). Adjuvanted Sc9-10-NPM induced a higher nAb response than the non-adjuvanted group, as expected, among which the MF59-biosimilar (MF59-bio) group induced the highest nAb titers. Under the same dosage (1 µg and 5 µg), Sc9-10-NPM/MF59-bio induced significantly higher nAb response against both RSV strains than the commercial vaccine Arevxy (except for the 5 µg group against B strain). Sc9-10-NPM/Alhydrogel induced a similar nAb titer to the control vaccine Arexvy, while the non-adjuvanted Sc9-10-NPM was less immunogenic than the control vaccine Arexvy. It is worth noting that at 1/5 of the control vaccine Arexvy’s dosage (i.e., 0.2 µg of Sc9-10-NPM vs. 1 µg of Arexvy), the nAb response induced by Sc9-10-NPM/MF59-bio was still significantly more than the control vaccine Arexvy. These results confirm that Sc9-10-NPM is a promising candidate for an RSV vaccine.

### Challenge of Sc9-10-NPM in cotton rats

**Fig 4:**
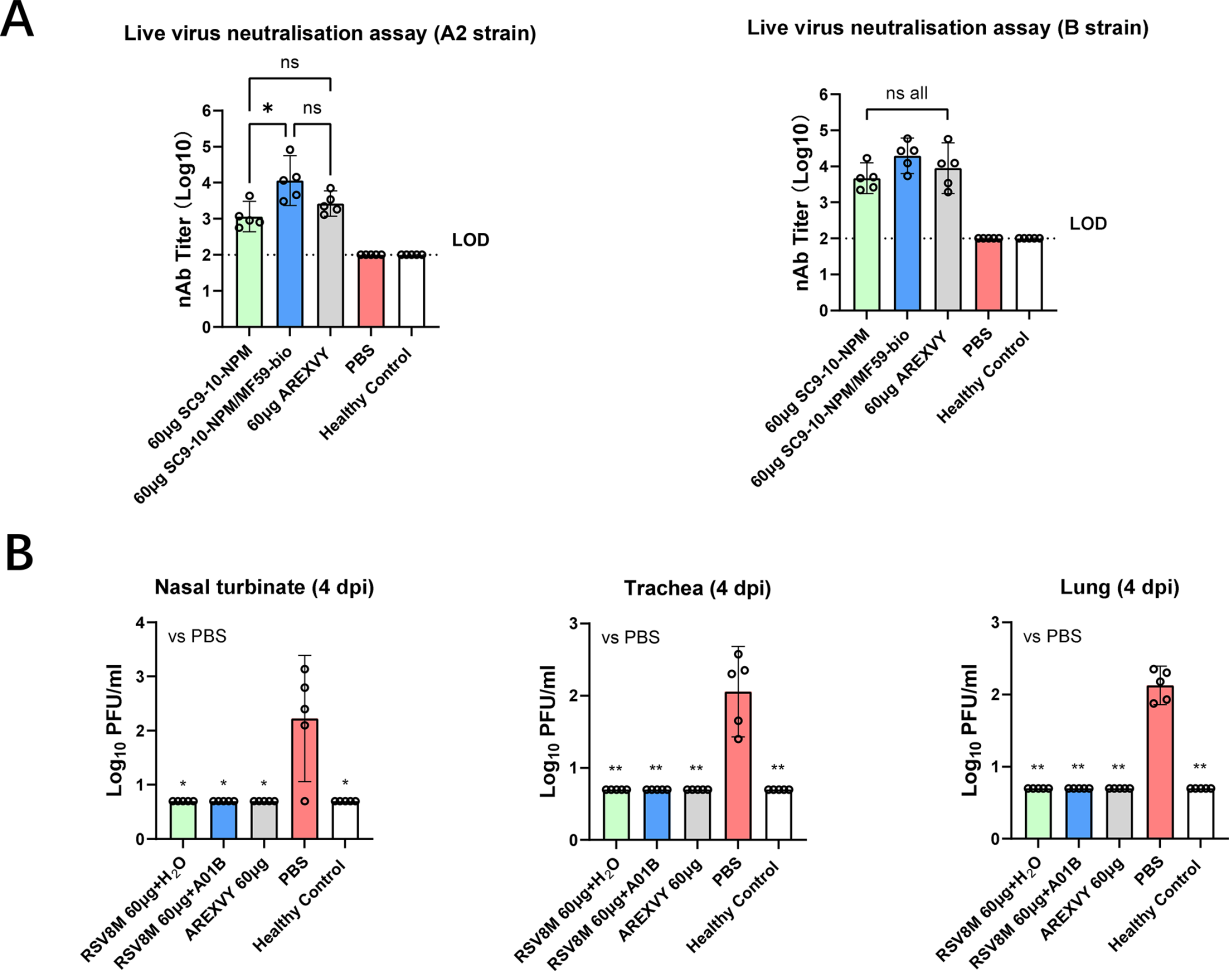
Challenge of SC9-10-NPM in cotton rats experiment #1. Male SPF-grade cotton rats (*n* = 5 per group) were immunized on day 0 and day 21 intramuscularly with either 60 µg of SC-9-10-NPM formulated in MF59-bio or 60 µg of control vaccine Arexvy. The PBS control and healthy animal control were included. Serum was collected on day 42 to measure neutralizing antibodies against RSV A2 and RSV B (**A**). RSV A2 (1.0 × 10^6^ PFU/animal) was challenged on day 42 via the intranasal route. Animals were sacrificed on day 46, and turbinate bones, trachea, and lung were collected to measure viral loads (**B**). GMT ± 95% CI is shown. Kruskal–Wallis test with Dunn’s multiple comparison test was performed, ns = not significant, *P < 0.05, **P < 0.01.

The first challenge experiment compared Sc9-10-NPM to the commercial vaccine Arexvy in terms of their effectiveness against viral challenge. Cotton rats were immunized twice with 60 µg Sc9-10-NPM formulated in MF59-bio adjuvant or in water (non-adjuvanted group), and with 60 µg (half human dose) of control vaccine Arexvy, on days 0 and 21. The PBS group and the healthy animal group were included as controls. Serum from each cotton rat was taken on day 41 for neutralizing antibody (nAb) assay. The cotton rats were then challenged with RSV/A2 (ATCC, VR-1540) live virus on day 42, followed by dissection on day 46. The viral loads in the turbinate bone, trachea, and lung tissue were measured by plaque assay. After two immunizations, Sc9-10-NPM formulated with MF59-bio adjuvant was able to induce a higher (but not significant) neutralizing antibody response against both RSV strains compared to the non-adjuvanted group and the control vaccine Arexvy (Fig. 4A). Even though the non-adjuvanted Sc9-10-NPM induced the lowest nAb response, it was not significantly different from the control vaccine Arexvy (Fig. 4A). After the challenge, we observed a substantial reduction in the viral load of all vaccine groups in the turbinate bone, trachea, and lung, compared to the PBS control (Fig. 4B). The results showed that all vaccine groups can provide complete protection against RSV in this animal model.

**Fig 5:**
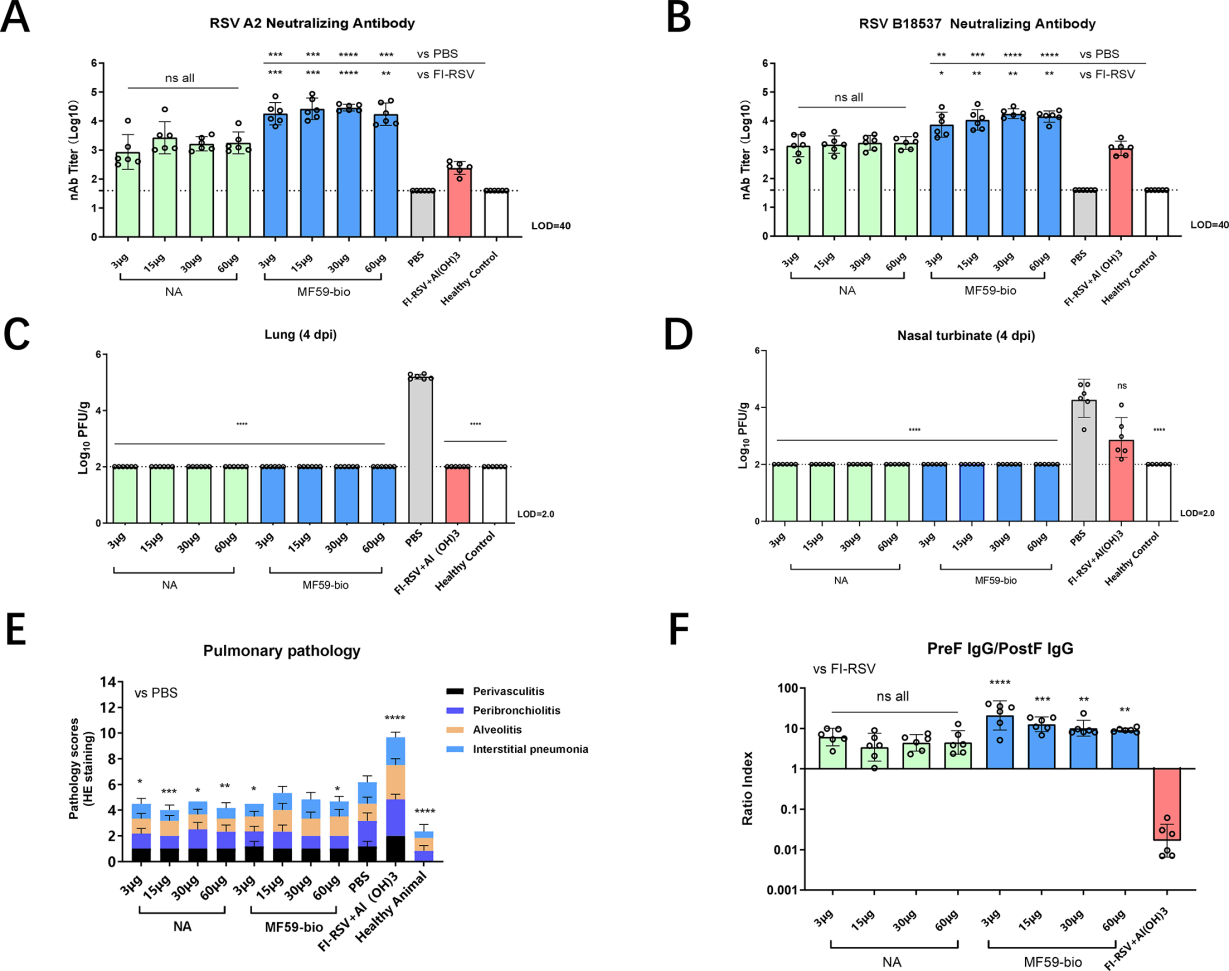
Challenge of SC9-10-NPM in cotton rats experiment #2. Male SPF-grade cotton rats (*n* = 6 per group) were immunized on day 0 and day 21 intramuscularly with 3 µg, 15 µg, 30 µg, or 60 µg of SC-9-10-NPM formulated either without adjuvant or in MF59-bio. The PBS control, healthy animal control, and the group receiving inactivated RSV formulated in aluminum hydroxide were included. Serum was collected on day 41 to measure neutralizing antibodies against RSV A2 (**A**) and RSV B18537 (**B**). RSV A2 (1.0 × 10^6^ PFU/animal) was challenged on day 42 via the intranasal route. Animals were sacrificed on day 46, and lung and turbinate bones were collected to measure viral loads (**C and D**). Pathology was measured and scored for individual lung tissues (vs PBS) (**E**), and PreF/PostF IgG ratio was also calculated (**F**). For (**A, B, C, D, and F**), GMT ± 95% CI is shown. Kruskal–Wallis test with Dunn’s multiple comparison test was performed, ns = not significant, *P < 0.05, **P < 0.01, ***P < 0.001, ****P < 0.0001. For (**E**), mean ± SD is shown. Ordinary one-way ANOVA was performed to compare each group with PBS control.

A second challenge experiment was performed to further analyze the effectiveness of Sc9-10-NPM in a broader dose range and to confirm the absence of the vaccine-enhanced disease (VED) response. Cotton rats were immunized twice on day 0 and day 21 with 3 µg, 15 µg, 30 µg, and 60 µg of Sc9-10-NPM formulated either in water (non-adjuvanted group) or in MF59-bio, and with formalin-inactivated RSV (FI-RSV) formulated in Alhydrogel. The PBS group and the healthy animal group were included as controls. Serum was collected from the cotton rat on day 41. Neutralizing antibody (nAb) was measured by microneutralization assay. The cotton rats were then challenged with RSV/A2 (ATCC, VR-1540) live virus on day 42, followed by dissection on day 46. The viral loads in the turbinate bone and lung tissue were measured by plaque assay. The lung tissue lesions were evaluated by HE staining, and histopathological effects were scored. After two immunizations, both the adjuvanted and non-adjuvanted groups of Sc-9-10-NPM (3, 15, 30, and 60 µg/animal) were able to induce higher nAb responses against RSV A2 and RSV B18537 than the FI-RSV group and PBS group. However, the difference was only significant in the adjuvanted group (Fig. 5A and B). No clear dose-dependent response was observed in the day 41 serum samples. After the challenge, all vaccine groups, including both adjuvanted and non-adjuvanted, as well as the FI-RSV group, demonstrated a significant reduction in viral load compared to the PBS group in the lung tissue (Fig. 5C). A similar trend was observed in the turbinate bone, except that the viral reduction in the FI-RSV group was not significant compared to the PBS control (Fig. 5D). Consistent with the first challenge study, the results showed that our vaccine groups can provide complete protection against RSV, even at low vaccination dosage. Lung tissue histopathology was analyzed and scored; no difference was observed between adjuvanted and non-adjuvanted groups, and all Sc-9-10-NPM groups showed similar or even lower scores than the PBS group. The FI-RSV group, on the other hand, had a significantly higher score than the PBS group (Fig. 5; Fig. S3). Furthermore, we analyzed the ratio of preF/postF IgG induced by all vaccine groups; it is evident that all Sc9-10-NPM groups induced a higher preF/postF profile than FI-RSV, notably only the adjuvanted group demonstrated a significant difference (Fig. 5F).

In conclusion, the Sc9-10-NPM candidate vaccines were effective across a wide dosage range in the cotton rat challenge model, regardless of the adjuvant used. No enhancement of lung pathology was observed, indicating that the designed nanoparticle displaying preF antigen could avoid the VED effect as observed in FI-RSV.

## DISCUSSION

RSV infections are widespread, particularly among infants, the elderly, and immunocompromised individuals. However, there are few therapeutic drugs or vaccines targeting RSV. The development of an RSV vaccine has been a lengthy process. It was not until 2013 that Peter Kwong’s group made a breakthrough by successfully generating a stable pre-fusion conformational mutant of the RSV F glycoprotein, known as DS-Cav1 (8). Building on this achievement, the Sc9-10 molecule was subsequently engineered to enhance stability and immunogenicity by using iterative cycles of structure-based vaccine design. These discoveries provided a new direction for the development of the RSV vaccine (9).

Based on this DS-Cav1 preF design and its derivatives, RSV vaccines have been developed and approved for use, including GSK’s subunit vaccine, Arexvy, Pfizer’s bivalent subunit vaccine, Abrysvo, and Moderna’s mRNA vaccine, mResvia. Arexvy and mResvia are approved for use only in adults aged 60 and above (10, 11, 20). Arexvy is not recommended for infants and young children, possibly due to the AS01E adjuvant (21, 22), nor for pregnant women due to the potential risk of preterm birth. Clinical trials of mResvia in pregnant women are ongoing; however, the FDA recently halted trials of mResvia in infants and young children due to the potential risk of antibody-dependent enhancement (ADE). Abrysvo, on the other hand, due to its better safety profile (non-adjuvant formulation) and subsequent efficacy data, is approved for use in adults aged 60 and above, as well as in pregnant women between 32 and 36 weeks of gestation. It can protect infants from birth to 6 months of age against RSV-related lower respiratory tract disease and severe lower respiratory tract disease (23).

To develop a new RSV vaccine candidate, one of our key considerations was to optimize its immunogenicity and durability by introducing a novel technological approach: nanoparticle vaccines. In recent years, recombinant self-assembling nanoparticles have undergone rapid development. They are considered a powerful platform for antigen display, as they can elicit stronger and longer-lasting humoral immunity through multivalent presentation while ensuring safety due to the lack of nucleic acids (24, 25). The King group first combined the DS-Cav1 with the trimeric I53-50A subunits through genetic fusion and subsequently achieved presentation of DS-Cav1 by assembling it with the I53-50B pentamer. This strategy significantly improved the immunogenicity of DS-Cav1. This advancement has important implications for the development of effective and safe vaccines using recombinant self-assembling nanoparticles (14). In this study, we aimed to display the RSV preF antigens on a nanoparticle Mi3 (NPM) platform. NPM has previously been proven to be a suitable platform for antigen presentation, demonstrating its efficacy in the protection against SARS-CoV-2 and Herpes zoster (26, 27). The Catcher/Tag system was employed to enhance adaptability and accessibility, allowing various tag-fused antigens to be readily assembled onto the surface of Catcher-modified NPM. Both DS-Cav1 and Sc9-10 were successfully conjugated to the NPM as confirmed through a series of assays, including SDS-PAGE, SEC, DLS, and negative-stained TEM. Both of the RSV preF antigens displayed on the NPM exhibited a higher affinity for the pre-fusion-specific antibody D25 compared to their soluble trimeric forms or even their conjugates on the I53-50 NP platform. Furthermore, consistent with previous reported studies, Sc9-10 demonstrated greater antigenicity than DS-Cav1 by exhibiting a stronger affinity for the neutralizing antibody D25 and a lower affinity to the post-fusion specific antibody 4D7, confirming its advantage over the first-generation antigen DS-Cav1. Subsequent antigenicity and immunogenicity studies confirmed Sc9-10-NPM to be the best candidate among all test groups. We also assessed the stability of four nanoparticles: DS-Cav1-NPM, Sc9-10-NPM, DS-Cav1-I53-50, and Sc9-10-I53-50. These nanoparticles were tested at −80°C for six months and at 4°C, 25°C, and 37°C for four weeks. All four nanoparticles exhibited excellent stability at 4°C and −80°C. However, when the temperature exceeded 25°C, both I53-50 nanoparticles, DS-Cav1-I53-50 and Sc9-10-I53-50, began to degrade quickly. In summary, the Sc9-10-NPM vaccine candidate exhibits exceptional performance in both enhancing the immunogenicity of RSV preF and maintaining antigen stability. The structural integrity and thermal stability of Sc9-10-NPM were measured and confirmed by AUC and DSC. It is also worth noting that our DSC analysis showed a double-peak thermograph, with the first and second melting temperatures (*T*_m_) determined at 62.3°C and 82.0°C, respectively. This observation was nearly identical to the *T*_*m*_ data of the original Sc9-10 reported previously, where two *T*_*m*_ temperatures peaked at 63.7°C and 82.2°C. The two distinct *T*_m_ values likely reflect a two-step unfolding process. The first *T*_m_, typically observed around 48°C–51°C, corresponds to the unfolding of relatively less stable and flexible regions, such as the F₂ subunit and the F₂–F₁ linker. The second higher *T*_m_, ranging from approximately 62°C to 72°C depending on the construct, represents the denaturation of the central trimeric core, including heptad repeat regions and interprotomer interfaces stabilized through structure-guided mutations (e.g., proline substitutions and interprotomer disulfide bonds). For example, DS-Cav1 exhibits two *T*_m_ peaks at ~48.6°C and ~71.7°C, while further stabilized variants, such as Sc9-10, show an increase in both *T*_m_ values, confirming enhanced prefusion conformational stability (28). Lastly, the BLI binding assay confirmed that Sc9-10 displayed on NPM retains its site Ø peptide and trimeric structure, demonstrating strong affinity for both site Ø-specific antibody D25 and quaternary-dependent antibody AM14. Therefore, the NPM platform successfully retained the biophysical properties of Sc9-10. Altogether, Sc9-10-NPM is a highly promising vaccine candidate with remarkable protective potential in vaccine research and development.

Another objective we consider when developing this new-generation RSV vaccine is to avoid the use of potent adjuvants, such as AS01E used in Arexvy, while maintaining a similar or higher level of efficacy. AS01E is known to be associated with adverse effects (AEs), especially the grade 3 AEs observed in Arexvy’s clinical trials (10). Sc9-10-NPM was subsequently compared head-to-head with the commercial vaccine Arexvy formulated in AS01E. In general, Sc9-10-NPM formulated with MF59-bio (a safer adjuvant compared to AS01E, as reviewed by Verstraeten et al. [29, 30]) induced higher neutralizing antibodies than Arexvy in both mice and cotton rats, while Sc9-10-NPM without adjuvant induced a lower nAb response. Nevertheless, all three groups exhibited similar levels of protection in two separate challenge studies. In a real-world scenario, the adjuvant may not be necessary due to pre-existing immunity. In published clinical trial data, several RSV vaccine candidates are as effective without the use of an adjuvant, although these data are contradicted by their preclinical studies (14, 31, 32). The lack of animal models representing the population with pre-existing immunity means that the necessity of using the adjuvant could only be confirmed by clinical trials. In our study, the observation of complete protection provided by our vaccine with a wide range from 3 μg to 60 μg per dose, both with and without adjuvant in the cotton rat models, supports further testing of both the adjuvanted and non-adjuvanted vaccine designs in clinical trials. In addition, we reported the absence of adverse VED effects in our vaccine, with or without adjuvant, as well as a higher preF/postF IgG ratio compared to FI-RSV, confirming the safety of the vaccine. Overall, our study demonstrated that the selected approach has the potential to lead to the development of a safe and effective vaccine against RSV.

## Data Availability

The data used in the current study are available from the corresponding authors upon reasonable request.

## SUPPLEMENTAL MATERIAL

Supplemental figures jvi.00903-25-s0001.pdf Fig. S1 to S3.

ASM does not own the copyrights to Supplemental Material that may be linked to, or accessed through, an article. The authors have granted ASM a non-exclusive, world-wide license to publish the Supplemental Material files. Please contact the corresponding author directly for reuse.
