# Supplementary material for: Nanoparticle vaccine based on the pre-fusion F glycoprotein of respiratory syncytial virus elicits robust protective immune responses
Source: J Virol. 2025 Aug 26;99(9):e00903-25. doi: 10.1128/jvi.00903-25 (PMC12455995; doi:10.1128/jvi.00903-25)
Supplement: Supplemental figures — Fig. S1 to S3. [file jvi.00903-25-s0001.pdf]

Nanoparticle vaccine based on the pre-fusion F glycoprotein of respiratory syncytial virus elicits robust protective immune responses

Zhulong Hu[1,7,#], Siyu Tian[1,#], Yu Zhou[1,#], Yanqun Wang[2,#], Yu Li[1], Senyan Zhang[1], Peilan Wei[2,3], Zhen Zhuang[2], Luo Ren[4,5], Jiao Liu[2], Na Zang[4], Rui Yu[1], Yanbin Ding[1], Yan Guo[1], Cai Jing[1], Hang Chen[1], Caixia Zhang[1], Yuanfeng Yao[1], Chunping Deng[1], Rui Wei[1], Peng Zhou[1], Yongjuan Zou[1], Dawei Zhao[1], Shuyun Liu[1], Meijuan Fu[1], Xuejun Mo[1], Guodong Peng[1], Enmei Liu[4,*], Jincun Zhao[2,3,6,*], Yuanyuan Li[1,*], Jing Jin[1,*]

Affiliation:

[1]Patronus Biotech Co. Ltd., Guangzhou, China

[2]State Key Laboratory of Respiratory Disease, National Clinical Research Center for Respiratory Disease, Guangzhou Institute of Respiratory Health, the First Affiliated Hospital of Guangzhou Medical University, Guangzhou, Guangdong, China

[3]Guangzhou National Laboratory, Bio-Island, Guangzhou, China

[4]Department of Respiratory Medicine, Children's Hospital of Chongqing Medical University, National Clinical Research Center for Child Health and Disorders, Ministry of Education Key Laboratory of Child Development and Disorders, Chongqing Key Laboratory of Pediatrics, Chongqing, China

[5]Pediatric Research Institute, Children's Hospital of Chongqing Medical University, Chongqing, China

[6]Shanghai Institute for Advanced Immunochemical Studies, School of Life Science and Technology, ShanghaiTech University, Shanghai, China

[7]State Key Laboratory of Biocatalysis and Enzyme Engineering, School of Life Sciences, Hubei University, Wuhan, China.

[#] These authors contributed equally

*Correspondence to: jinjing@luye.com ; liyuanyuan@luye.com ; zhaojincun@gird.cn ; emliu186@126.com

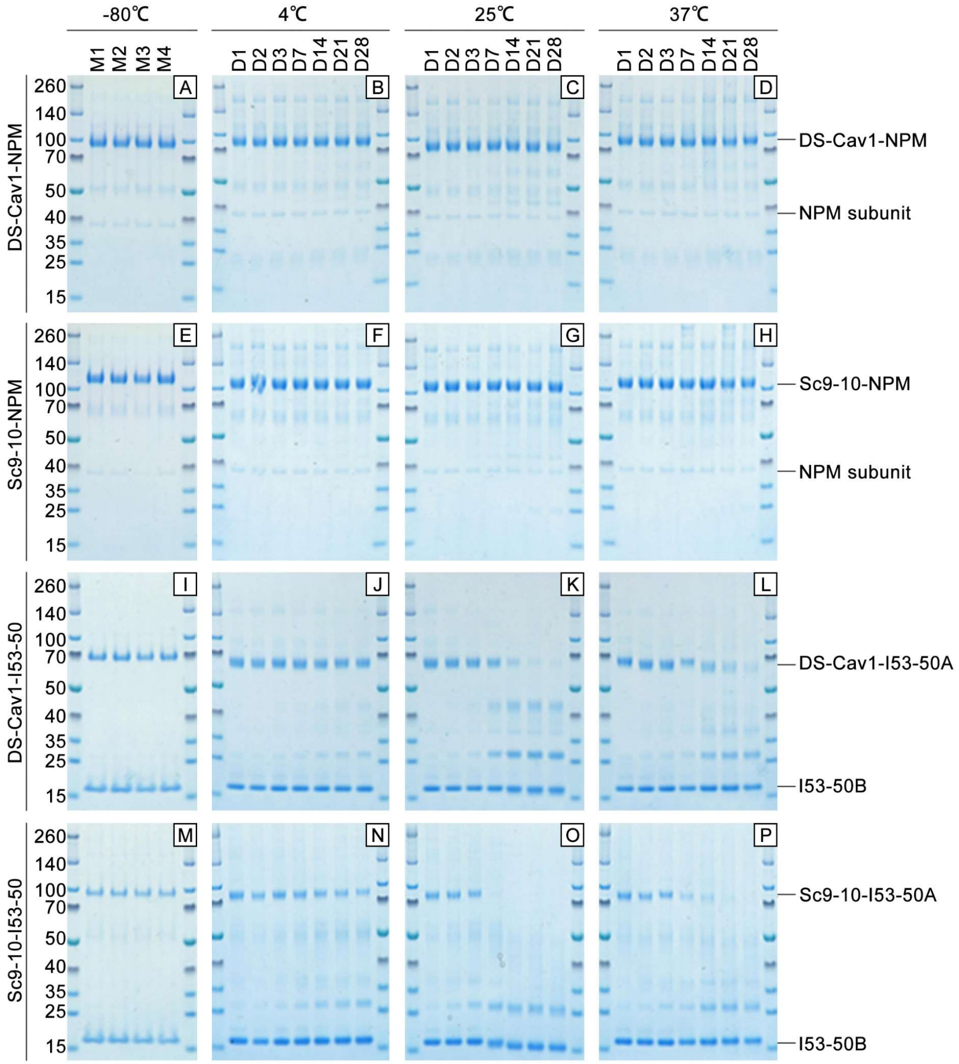

Figure S1. Stability of candidate vaccines.
After purification, vaccine candidates were aliquoted and stored under -80°C, 4°C, 25°C and 37°C, respectively. Samples from different time points were assessed by SDS-PAGE under reducing condition. The protein ladder's molecular weight and the sample bands' identity are labelled.

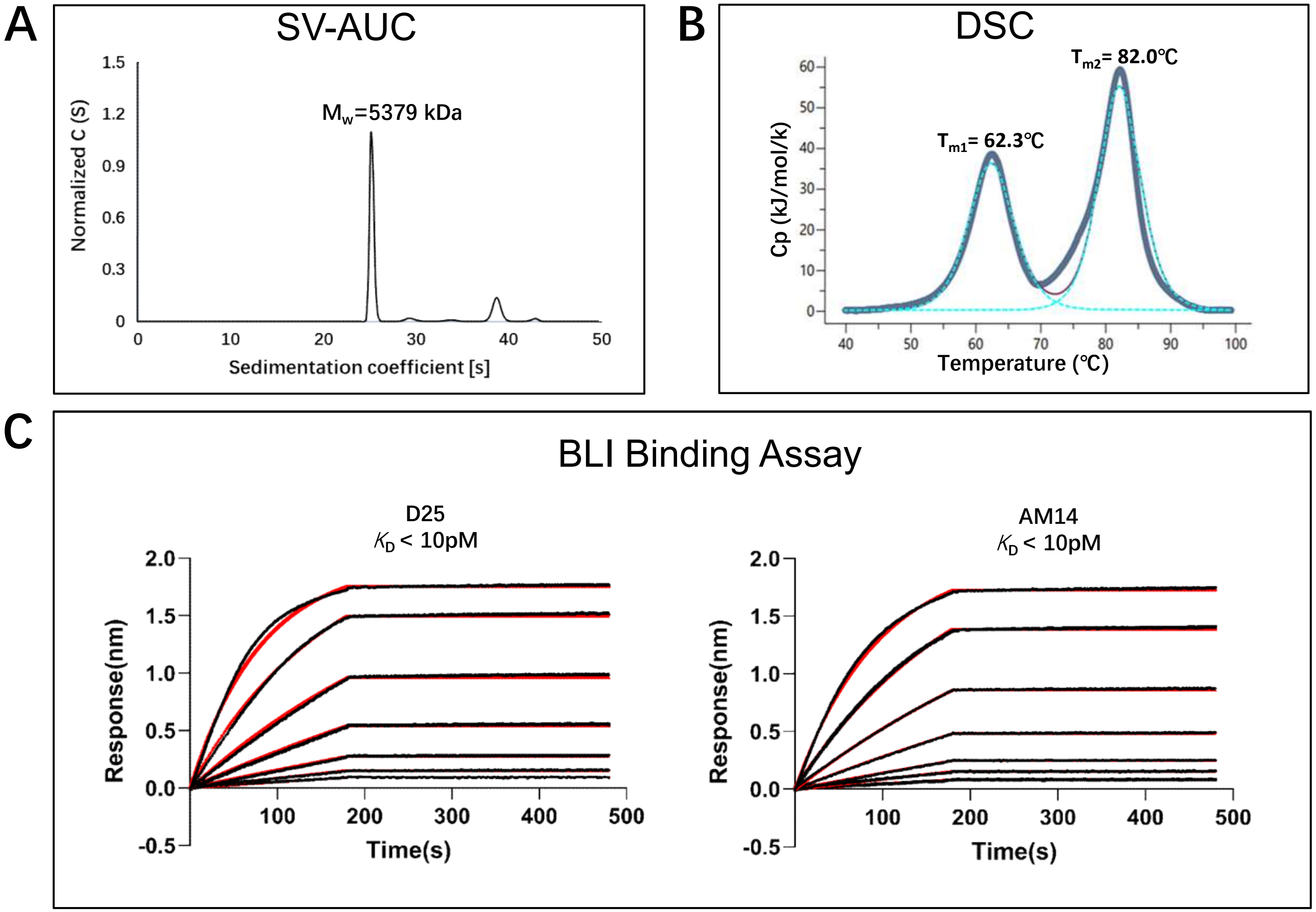

Figure S2. Biophysical and epitopes characterization of SC9-10-NPM.
SC9-10-NPM was further measured for molar mass and sedimentation profile by SV-AUC **(A)**, temperature stability profile by DSC **(B)** and the binding kinetics to the pre-fusion-specific antibody D25 (left panel); Binding of quaternary specific antibody AM14 (right panel), K_D **<** 10pM indicating high binding affinity **(C)**. Abbreviations: SV-AUC, Sedimentation velocity analytical ultracentrifugation; DSC, differential scanning calorimetry; BLI, Biolayer interferometry.

The lung tissue HE staining (×100)

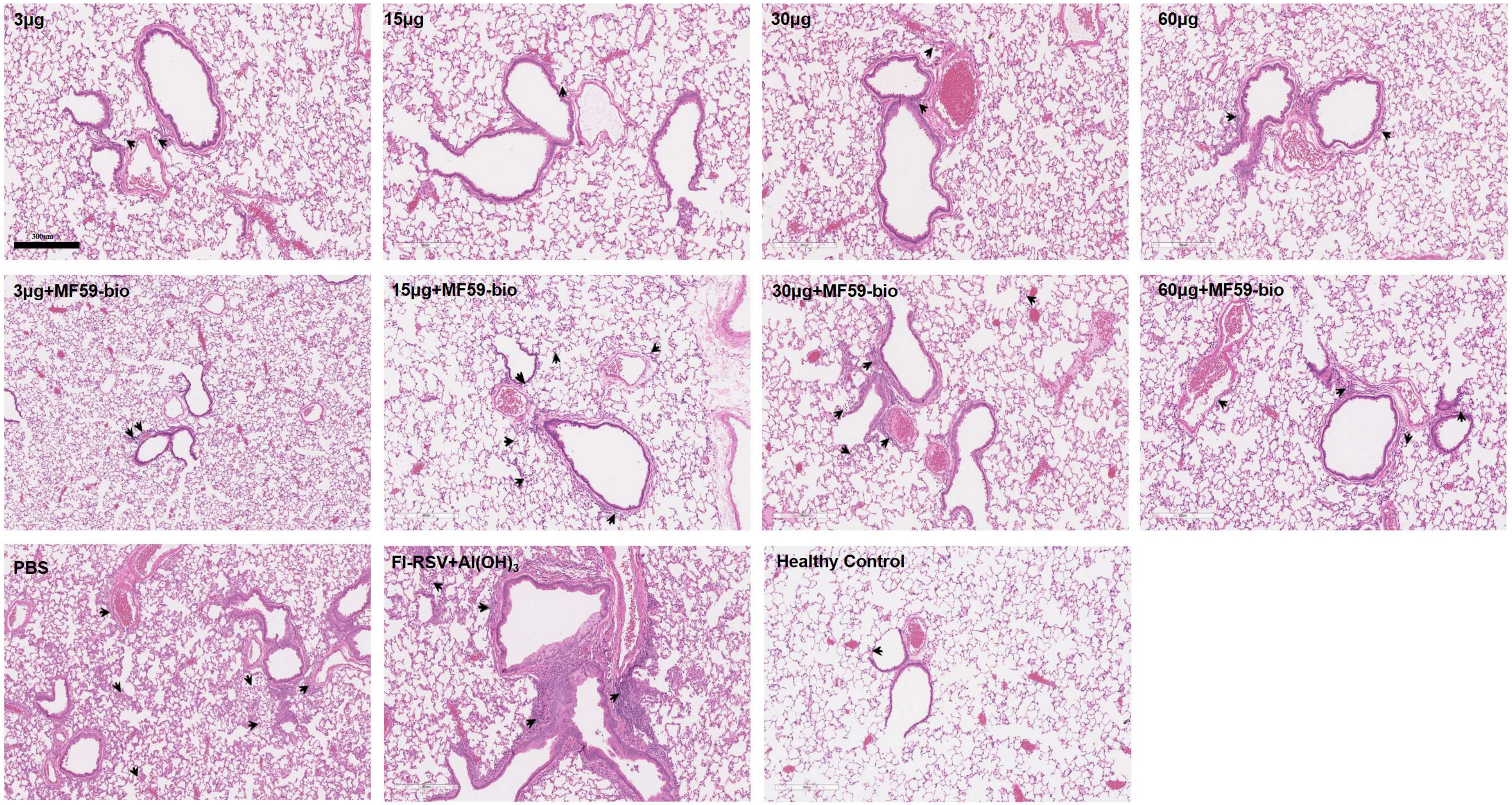

Figure S3. Histopathological analysis of lung

Lung tissues were harvested at 4 dpi. Sections were stained with H&E (n = 6 mice per group). Arrowhead indicates regions with interstitial pneumonia, alveolitis, peribronchiolitis, and perivasculitis. Representative pathological images were from six independent mice per group. Scale bars (black), 300 μm.
